# Examining the causal mediating role of brain pathology on the relationship between diabetes and cognitive impairment: the Cardiovascular Health Study

**DOI:** 10.1111/rssa.12570

**Published:** 2020-05-08

**Authors:** Ryan M. Andrews, Ilya Shpitser, Oscar Lopez, William T. Longstreth, Paulo H. M. Chaves, Lewis Kuller, Michelle C. Carlson

**Affiliations:** Johns Hopkins University, Baltimore, USA, and Leibniz Institute for Prevention Research and Epidemiology—BIPS, Bremen, Germany; Johns Hopkins University, Baltimore, USA; University of Pittsburgh School of Medicine, USA; University of Washington, Seattle, USA; Florida International University, Miami, USA; University of Pittsburgh, USA; Johns Hopkins University, Baltimore, and Johns Hopkins Center on Aging and Health, Baltimore, USA

**Keywords:** Alzheimer’s disease, Causal inference, Causal mediation analysis, Cognitive neuroscience, Public health

## Abstract

The paper examines whether *diabetes mellitus* leads to incident mild cognitive impairment and dementia through brain hypoperfusion and white matter disease. We performed inverse odds ratio weighted causal mediation analyses to decompose the effect of diabetes on cognitive impairment into direct and indirect effects, and we found that approximately a third of the total effect of diabetes is mediated through vascular-related brain pathology. Our findings lend support for a common aetiological hypothesis regarding incident cognitive impairment, which is that diabetes increases the risk of clinical cognitive impairment in part by impacting the vasculature of the brain.

## 1. Introduction

*Diabetes mellitus* is a cluster of progressive metabolic diseases that typically develop in midlife from inadequate insulin secretion or insulin action, which leads to hyperglycaemia and possibly long-term damage to multiple organs, including the blood vessels (American Diabetes Association, 2014). World wide, approximately 8–14% of the population has diabetes, with the highest prevalence seen among non-Hispanic black and Hispanic populations (Menke et al., 2015; Tamayo et al., 2014). Prevalence estimates rise to 20–33% among those age 65 years or older (Kirkman et al., 2012). *Diabetes mellitus* (otherwise known as type 2 diabetes) is by far the most common type of diabetes among middle-aged and older adults (American Diabetes Association, 2014; Ogurtsova et al., 2017).

Given that up to a third of older adults have *diabetes mellitus* and that the prevalence of the disease is expected to rise over the next few decades (Kirkman et al., 2012; Menke et al., 2015; Ogurtsova et al., 2017), an important public health question is to understand how and why it impacts multiple systems and various health-related outcomes, including cognition. Prior studies have shown an association between *diabetes mellitus* and a number of adverse cognitive outcomes, including impaired baseline cognition, accelerated cognitive decline and higher risk of both mild cognitive impairment (MCI) and Alzheimer’s disease (AD), even after accounting for common risk factors like age, obesity, level of education and comorbidities (Bangen et al., 2015; Cukierman et al., 2005; Gregg et al., 2000; Mayeda et al., 2015; Palta et al., 2017; Yaffe et al., 2011). In fact, some consider AD to be ‘type 3’ diabetes, given the strong association between the two disorders (de la Monte, 2014; Kandimalla et al., 2017; Leszek et al., 2017; Steen et al., 2005).

One hypothesis for why diabetes increases the risk of cognitive impairment is that it leads to vascular brain injuries, such as brain hypoperfusion and white matter disease, which in turn lead to cognitive impairment. In other words, the effect of diabetes on cognitive impairment may be mediated through cerebrovascular pathology. Support for this hypothesis comes from several observational studies finding that type 2 diabetics have increased risk of stroke (Carson et al., 2012; Khoury et al., 2013; Mortel et al., 1990; Ottenbacher et al., 2004), brain hypoperfusion (Falvey et al., 2013; van Harten et al., 2006) and white matter disease (de Bresser et al., 2010; Reijmer et al., 2013). All these vascular risk factors have been shown to be strongly related to incident cognitive impairment (Gorelick et al., 2011; Kuller et al., 2004; Troncoso et al., 2008) in line with the ‘vascular’ hypothesis of Alzheimer’s disease (de la Torre, 2012; de la Torre and Mussivand, 1993). There have also been molecular biology and animal studies that provide experimental evidence suggesting that cardiovascular disease lies along a causal pathway from *diabetes mellitus* to cognitive impairment. For example, Zuloaga et al. (2016) randomized mice to either a high fat diet designed to model *diabetes mellitus* or a low fat control diet, and then half of the mice in each group received a surgical intervention that results in brain hypoperfusion. They found that both high fat diet and brain hypoperfusion increased the risk of cognitive impairment in mice, but that the high fat diet’s effect on cognitive impairment could not be explained by brain hypoperfusion alone. These controlled animal models lend further support to the potential for both a direct and an indirect causal effect between *diabetes mellitus* and cognitive impairment through cerebrovascular brain pathology. Several other animal model studies have found comparable results (Carvalho et al., 2013; Taguchi, 2009).

Two gaps that were highlighted by the studies above, however, are

- that, to our knowledge, there has not been any human population-based study that addresses the question of whether cerebrovascular pathology is a mediator of the diabetes–cognitive impairment relationship and
- whether these relationships are causal.

With respect to the first shortcoming, several studies have shown that cerebrovascular factors are associated with increased risk of cognitive impairment among type 2 diabetics (Bruce et al., 2008; Manschot et al., 2006; Umegaki et al., 2008, 2015); however, these studies lack the temporal ordering that is needed to investigate whether type 2 diabetics have increased risk for cognitive impairment because *diabetes mellitus* and cognition share common causes or risk factors, or whether *diabetes mellitus* directly or indirectly leads to AD-related pathological changes like brain atrophy, neuronal death and plaques and tangles. Wang et al. (2017) presented results that suggest that the effect of cardiovascular burden (including diabetes) on cognitive decline is mediated by brain pathology; however, they did not consider whether the same relationships hold with respect to cognitive impairment. With regard to the second gap, it is impossible to assign *diabetes mellitus* randomly to individuals in a study for ethical reasons, which means that any study of a causal mechanism must rely on observational data. This restriction typically leads clinical and public health researchers to be circumspect with their conclusions, discussing them in terms of associations or correlations even though what they truly aim to estimate is a causal relationship (see Hernán (2018) for a discussion).

In this paper, our goal is to assess the relative magnitude of the indirect causal effect of diabetes among older adults on incident MCI or dementia through two markers of vascular brain injury: brain hypoperfusion and white matter disease. The data set that was used to address these questions was the Cardiovascular Health Study (CHS): a large 10-year prospective study of US older adults that was designed to study cardiovascular risk factors in later life through clinically adjudicated cognitive outcomes and brain and blood biomarkers (Fried et al., 1991; Lopez, Kuller, Fitzpatrick, Ives, Becker and Beauchamp, 2003). To decompose the potential effect of diabetes on cognitive impairment, we used the inverse odds ratio weighting (IORW) approach of Tchetgen Tchetgen (2013), which enables the estimation of direct and indirect effects that can be interpreted as causal, provided that certain assumptions are met. Our results show that, under these assumptions, brain hypoperfusion and white matter disease jointly account for approximately a third of the total effect of diabetes on cognitive impairment.

The paper is organized as follows. Section 2 describes the CHS and our sample selection procedure. Section 3 details our estimation strategy, including the details of our causal mediation analysis and the assumptions on which it relies. Section 4 presents our results. Finally, Section 5 concludes the paper with a discussion of our findings.

## 2. Data and sample selection

### 2.1. The Cardiovascular Health Study

The data for our analysis come from the CHS, which is a population-based sample of 5888 adults age 65 years or older who reside in one of four regions in the USA:

- Forsyth County, North Carolina,
- Sacramento County, California,
- Washington County, Maryland, or
- Pittsburgh, Pennsylvania.

The recruitment strategy, study design and eligibility criteria of the CHS have been described elsewhere (see Tell et al. (1993) and Fried et al. (1991)). In short, the CHS initially recruited adults in 1989–1990 from a random sample of all Medicare eligible individuals living in census tracts near the four recruitment regions, and a racial minority supplement of 687 individuals was added in 1992–1993. All participants were asked to complete annual in-person interviews and clinical assessments from baseline until 1998–1999. In addition to the data that were provided by the participants, the CHS also gathered information on all participant hospitalizations, including international classification of diseases, ninth revision, codes and discharge summaries. All CHS participants provided written informed consent, and the institutional review boards at each participating location approved the CHS study procedures.

We restricted our analytic sample to those in the CHS Cognition Study, which was an ancillary study to the main CHS designed in 1999 to investigate risk factors for dementia and MCI. The details of the cognition study have been published elsewhere (Lopez, Kuller, Fitzpatrick, Ives, Becker and Beauchamp, 2003). In brief, all CHS participants were eligible for the cognition study if they completed a magnetic resonance imaging (MRI) examination between 1991 and 1994 and a modified mini mental status examination evaluation, which is known as ‘3MS’. This resulted in a possible sample of 3602 participants (Lopez, Kuller, Fitzpatrick, Ives, Becker and Beauchamp, 2003).

### 2.2. Diabetes mellitus

At the baseline visit, fasting blood was drawn, kept frozen at −70 °C and shipped to a central blood analysis laboratory where serum analyses were carried out on a Kodak Ektachem 700 Analyzer (Fried et al., 1991; Irie et al., 2008). Fasting glucose and insulin levels were assayed, using solid phase radioimmunoassay and serum-based standards (Irie et al., 2008). Baseline diabetes was defined as a fasting glucose concentration of 126 mg dl^−1^ or higher, or the reported use of hypoglycaemic medication or insulin treatment. Participants who were determined to be prediabetic were considered non-diabetic for this analysis.

### 2.3. Clinical cognitive impairment

For our study, the primary outcome of clinical cognitive impairment was a diagnosis of either MCI or dementia, which were adjudicated by a panel of clinical and content experts (Lopez, Jagust, DeKosky, Becker, Fitzpatrick, Dulberg, Breitner, Lyketsos, Jones, Kawas, Carlson and Kuller, 2003; Lopez et al., 2005; Lopez, Kuller, Fitzpatrick, Ives, Becker and Beauchamp, 2003). In 1999 at the end of the clinic visits for CHS, individuals were retrospectively adjudicated for dementia starting from the date of their first MRI scan through to the end of the study. Diagnoses were made using the participants’ cognitive examination, assessment of functional status, proxy interviews, physician questionnaires, telephone interviews and medical chart review, and all participants (including those who died during the study) were adjudicated if they met the inclusion criteria for the cognition study. Dementia diagnostic criteria were based on impairments in two or more cognitive domains and a history of normal intellectual function, whereas MCI diagnostic criteria were based on impairments in at least one cognitive domain, subjective cognitive complaints (either by the participant or by the participant’s family members) and the absence of neurological, psychiatric or systemic illnesses that could explain the presence of cognitive deficits. Following other CHS dementia studies (Irie et al., 2008; Lyketsos et al., 2002; Podewils et al., 2005), MCI and dementia outcomes were merged into a single diagnostic entity.

### 2.4. Brain hypoperfusion and white matter disease

We considered two markers of vascular brain injury as mediators: brain hypoperfusion and white matter disease. Brain hypoperfusion was defined as one or more of incident stroke, incident transient ischaemic attack or MRI findings of infarcts or extensive white matter signal abnormality. When a stroke or transient ischaemic attack was reported during follow-up, a provisional diagnosis was assigned by the relevant CHS field investigator, reviewed by the CHS co-ordinating centre and finally reviewed and adjudicated at annual meetings of the CHS Morbidity Review Committee (Longstreth et al., 2001). Brain infarcts were identified on brain MRI and were defined on the basis of MRI signal characteristics and size 3 mm or more (Bernick et al., 2001; Bryan et al., 1994).

The presence of white matter disease was defined by using a semiquantitative 10-point white matter grading scale developed in the CHS (Manolio et al., 1994; Yue et al., 1997) designed to estimate the total extent of periventricular and subcortical white matter signal abnormality. A score of 0 or 1 represented no or barely detectable change in white matter signal, whereas a score of 9 indicated that nearly all the white matter was involved. Following prior CHS studies, we dichotomized participants as having extensive white matter disease if their white matter grade was between 3 and 9 (see Kuller et al. (2004, 2016) and Rosano et al. (2015)).

### 2.5. Covariates

In these analyses, we adjusted for possible confounding factors, including age, race, male sex, total cholesterol level, level of education, the presence of one or more apolipoprotein E4 (allele APOE-4), smoking status, hypertension, body mass index and antihypertensive medication use. All confounding factors were considered at their baseline values. Race was treated as a binary variable (white *versus* non-white), and APOE-4 status was also binary based on the presence of at least one allele. Smoking status was defined as current, former or never smoked. Hypertension was defined by systolic blood pressure of at least 140 mm Hg or diastolic blood pressure of at least 90 mm Hg, and antihypertensive medication use was defined as taking any antihypertensive medication. Following related prior work in the CHS (see Armstrong et al. (2017, 2018)), education was dichotomized as having completed high school *versus* not. All other confounding variables were treated as continuous.

### 2.6. Final analytic sample

#### 2.6.1. Primary analysis

Because we sought to examine how diabetes impacts incident MCI and dementia through brain hypoperfusion, we excluded any CHS Cognition Study participants who had prevalent dementia (*N* = 227) or a history of stroke or transient ischaemic attack (*N* = 94) at baseline. In addition, we excluded participants who did not have a baseline diabetic status (*N* = 24). Finally, we excluded any participant who developed MCI or dementia within 3 years of baseline (*N* = 32), to avoid misspecifying the temporal order of events for our mediation analysis (i.e. ensuring that brain hypoperfusion and white matter disease precedes MCI or dementia). Thus, our eligible sample size was 3225 individuals. In addition, excluding individuals with missing data on one or more covariates resulted in a final analytic sample of 2872 participants. The 10% who were excluded for missing data were primarily missing APOE-4 status (268 out of 332, or 80.7%) and were statistically comparable with those who had complete data.

#### 2.6.2. Secondary analysis

In addition to the primary analysis, we conducted two secondary analyses where we limited the outcome to either MCI or dementia rather than their combination. For the analysis involving MCI only, we excluded those who were classified with incident dementia after all other exclusion criteria had been applied (*N* = 442) and, for the analysis involving dementia only, we excluded MCI cases (*N* = 541) after all other exclusion criteria had been applied. This resulted in smaller eligible sample sizes: 2783 for the MCI analysis and 2684 for the dementia analysis. After removing individuals with missing data, our analytic sample sizes for the secondary analyses were 2527 for MCI and 2409 for dementia.

## 3. Estimation strategy

### 3.1. Counterfactual notation and average causal effects

We are interested in estimating the causal effect of *diabetes mellitus* on cognitive impairment. To do this, we utilize the counterfactual notation of Neyman (1923) and Rubin (1974). Assume that our sample consists of *N* individuals, whom we can index by the subscript *i* = 1,…, *N*, and, for each individual, we denote his diabetic status by *A*_*i*_ = 1 for diabetic and *A*_*i*_ = 0 for non-diabetic. In this paper, we refer to *A*_*i*_ interchangeably as either a treatment or exposure. Furthermore, let *Y*_*i*_ denote an individual’s cognitive impairment status, with *Y*_*i*_ = 1 indicating cognitive impairment and *Y*_*i*_ = 0 indicating no impairment, and assume that we collected a vector of covariate information for each individual, denoted by *C*_*i*_. We can define an individual’s *counterfactual outcome* as *Y*_*i*_(*A*_*i*_ = *a*), which in our case defines what an individual’s cognitive impairment status would be if he had (possibly counter to fact) had diabetes (i.e. *Y*_*i*_(*A*_*i*_ = 1)) or if he had (possibly counter to fact) not had diabetes (i.e. *Y*_*i*_(*A*_*i*_ = 0)). A causal effect of *A*_*i*_ on *Y*_*i*_ exists if *Y_i_*(*A_i_* = 1) ≠ *Y_i_*(*A_i_* = 0). Under the assumption that sampled individuals are independent and identically distributed, we treat *A*, *Y* and *C* as random variables and suppress the index *i*. Obviously, an individual either has or does not have *diabetes mellitus* in real life, which means that both *Y*(*A* = 1) and *Y*(*A* = 0) cannot be observed in real data. This leads many researchers to focus on population level causal effects rather than individual level effects, and we say that an average causal effect (ACE) exists in a population if *E*{*Y*(*A* = 1)} ≠ *E*{*Y*(*A* = 0)}, where *E* is the expectation operator. More generally, the ACE can be defined as

(1) $$\text{ACE}=h^{-1}[E\{Y(1)\}]-h^{-1}[E\{Y(0)\}]$$

where *h*^−1^ is a user-specified link function. If equation (1) is non-zero, this indicates the existence of an effect of the exposure on the outcome. When *h*^−1^ is the identity link, equation (1) is simply a linear contrast of conditional expectations; however, equation (1) also enables the ACE to be estimated by generalized linear models with non-linear link functions, like logistic regression.

To identify the ACE by using observed data, it suffices to consider four assumptions. The first is the consistency assumption, which states that, if *A* = *a*, then *Y*(*a*) = *Y* or, in other words, that the observed outcome *Y* when *A* = *a* is equal to the counterfactual outcome *Y*(*a*) (Cole and Frangakis, 2009; Pearl, 2010; VanderWeele, 2009). The second assumption is conditional exchangeability, which states that *Y*(*a*) ⊥ *A*∣*C* for all *A*. In other words, counterfactual outcomes are independent of exposure within levels of covariates *C*. Implicit in this assumption is also the assumption of no measurement error. The third assumption is positivity, which requires that, for a discrete exposure *A* and confounder vector **C**, if *f*(*C*) ≠ 0 then Pr(*A* = *a*∣**C**) > 0 for all possible values of *A*, where *f*(**C**) is the probability density function of **C** (Hernan and Robins, 2006; Westreich and Cole, 2010). In essence, this means that, within every combination of observed confounders, there are both exposed and unexposed individuals. Finally, we assume non-interference between individuals (Hudgens and Halloran, 2008; Ogburn and VanderWeele, 2014). This means that we assume that one individual’s counterfactual outcomes are unaffected by another individual’s treatment assignment. Under these assumptions, the ACE can be validly estimated by using any number of approaches, including regression analysis, provided that the exposure–outcome relationship is correctly modelled.

### 3.2. Natural direct and indirect effects

Because we were interested in studying two possible mediators on the causal pathway between *diabetes mellitus* and cognitive impairment, we define the counterfactual value of the mediator to be *M*(*a*) = {*M*_1_(*a*), *M*_2_(*a*)}, with an intuitive meaning that is similar to *Y*(*a*) defined earlier and *M*_1_ and *M*_2_ denoting brain hypoperfusion and white matter disease respectively. For example, in our study, *M*(1) represents the value of *M* that would be observed if an individual had been (possibly counter to fact) a type 2 diabetic. When defining mediation effects, it is also common to use counterfactuals of the form *Y*{*a*, *M*(*a*)} in which the treatment and the mediator are ‘nested’ together. This notation is useful, since it permits differing treatment assignments for the outcome and the mediator, e.g. *Y*{1, *M*(0)} for the counterfactual outcome in which *A* = 1 for the purposes of the outcome and *A* = 0 for the purposes of the mediator. Using this notation, we can define two effects—the natural direct effect (NDE) and the natural indirect effect (NIE). On the mean scale, the NDE and NIE can be written as

(2) $$\text{NDE}=h^{-1}(E[Y\{1,M(0)\}])-h^{-1}(E[Y\{0,M(0)\}]),$$

(3) $$\text{NIE}=h^{-1}(E[Y\{1,M(1)\}])-h^{-1}(E[Y\{1,M(0)\}]).$$

**Fig. 1.**
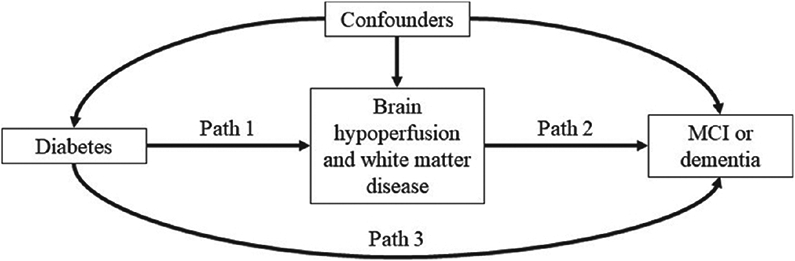
Directed acyclic graph of our mediation model: the indirect effect of diabetes on MCI or dementia through brain hypoperfusion and white matter disease (paths 1 and 2) and the direct effect of diabetes on MCI or dementia (path 3) will be estimated as part of the analysis

The ability for mediation effects to be identified without regard to exact model specification is a key advantage of the causal approach to mediation, compared with other approaches, like structural equation modelling, which rely on strong parametric assumptions for identification of effects (Shpitser, 2013). Intuitively, the NDE corresponds to the effect of the exposure on the outcome if the pathway through the mediator were somehow disabled (in Fig. 1, this would mean that paths 1 and 2 would be disabled), whereas the NIE corresponds to the effect of the exposure on the outcome if the pathway directly from the exposure to the outcome were disabled (in Fig. 1, this would mean that path 3 would be disabled). If there are no exposure–mediator interactions, the NDE and NIE sum to the ACE.

To identify these effects from observational data, it suffices to consider additional assumptions (Pearl, 2001, 2012; Robins and Greenland, 1992).

*Assumption 1. Y*(*a, m*) ⊥ *A*∣*C*.

*Assumption 2. M*(*a*) ⊥ *A*∣*C*.

*Assumption 3. Y*(*a, m*) ⊥ *M*∣*A, C*.

*Assumption 4. Y*(*a, m*) ⊥ *M*(*a′*)∣*C*.

**Fig. 2.**
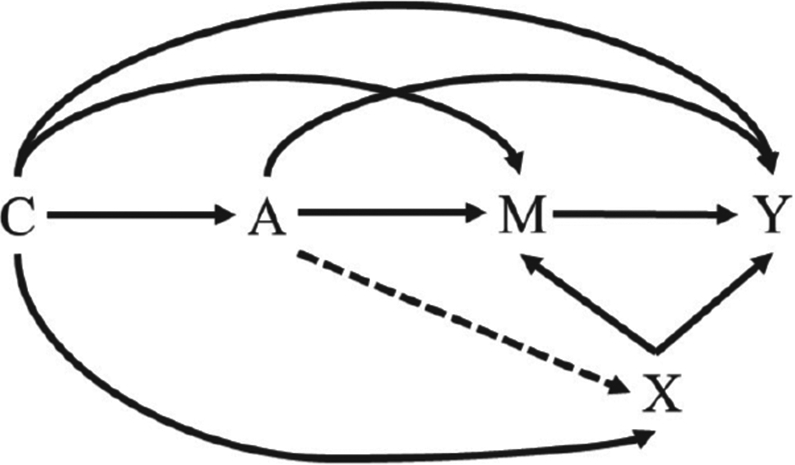
Hypothetical observed data graph in which the assumption of no mediator–outcome confounder that is affected by the exposure holds (- - - →, absence of an arrow): the letters *C* and *X* represent confounders, whereas the letter *A* denotes a treatment, *M* denotes a mediator and *Y* denotes an outcome

Assumptions 1–3 correspond to no unmeasured confounding of the treatment–outcome relationship, no unmeasured confounding of the treatment–mediator relationship and no unmeasured confounding of the mediator–outcome relationship respectively. The fourth assumption is arguably the least intuitive and corresponds to an assumption that there is an independence across counterfactual worlds, namely that the counterfactual for the outcome in a world where exposure is set to *A* = *a* is independent of the counterfactual for the mediator in a world where exposure is set to *A* = *a′*. This ‘cross-world’ assumption will be usually satisfied if there is no mediator–outcome confounder that is affected by exposure (Richardson and Robins, 2013). More generally, the cross-world assumption is satisfied if we view *Y*(*a, m, x*) and *M*(*a′ x′*) as structural equations *f*_*Y*_(*a, m, x, ϵ_Y_*) and *f*_*m*_(*a′, x′, ϵ_M_*) (where *X* is a possible mediator–outcome confounder) and have that *ϵ_Y_* and *ϵ_M_* are marginally independent regardless of how the inputs to the structural equations are set. This assumption is perhaps best understood by looking at a graph. In Fig. 2, we see that the variable *X* is a confounder of the mediator *M* and the outcome *Y*. The dotted arrow from the treatment *A* to *X* indicates that we are assuming that *A* does not affect *X*. If this arrow is present, we have a violation of this assumption because *ϵ_Y_* and *ϵ_M_* will not be marginally independent. Since this can never be verified or disproven experimentally, it is a strong assumption. However, we refer the reader to the discussion in Robins and Richardson (2010) for an intervention-based reconceptualization of mediation analysis under which this assumption could be tested. When satisfied, assumptions 1–4 are sufficient to allow for the estimation of NDEs and NIEs by using observed data and using convenient methods, including parametric regression. Since we are considering a joint mediator, we are assuming that there is a set of covariates *C* such that assumptions 1–4 are satisfied for brain hypoperfusion and white matter disease jointly.

### 3.3. Inverse odds ratio weighting

For our study, we estimated the NDE and NIE of *diabetes mellitus* on cognitive impairment through brain hypoperfusion and white matter disease by using IORW (Tchetgen Tchetgen, 2013). The IORW approach is a flexible framework for estimating mediation effects that can handle multiple mediators and can be used to decompose the total effect of an exposure into an NDE and NIE for most regression models that are used in policy or epidemiologic research (Nguyen et al., 2015). At a high level, this is done by first estimating the total effect of the exposure on the outcome, then estimating the NDE through a weighted regression and then estimating the NIE as the difference on the additive scale between the total effect and NDE (and then transforming these estimates back to their original scale, if applicable, like with logistic or Poisson regression). Formally, the method assumes that the total effect of *A* on *Y*, *γ*_tot_, can be estimated by fitting a mean regression model

$$h^{-1}[E\{Y|A=a,C=c;\psi \}]={\tilde{\gamma }}_{\text{tot}}(c;\psi_{\text{tot}})a+{\tilde{\gamma }}^{a^{\prime }}(c;\psi_{a^{\prime }})$$

where by consistency and conditional exchangeability ${\tilde{\gamma }}_{\text{tot}}(c;\psi_{\text{tot}})=\gamma_{\text{tot}}(c)$ is a parametric model for the total effect with unknown parameter *ψ*_tot_, ${\tilde{\gamma }}^{a^{\prime }}(c;\psi_{a^{\prime }})=h^{-1}[E\{Y(a^{\prime })|C\}]$ is a parametric model for the mean of *Y*(*a′*) with unknown parameter *ψ*_*a′*_ and $\psi =(\psi_{\text{tot}}^{T},\psi_{a^{\prime }}^{T})$. It further assumes that *ψ* can be estimated by $\hat{\psi }$, which solves the estimating equation

$$0=P_{n}[\Delta_{\text{tot}}(A,C;\hat{\psi })\{Y-E(Y|A=a,C=c;\hat{\psi })\}]$$

where Δ_tot_ is a vector of size dim(*ψ*) and *P_n_* denotes the empirical average operator. A convenient estimator for Δ_tot_ (*a, c*; *ψ*) is the maximum likelihood estimator. Furthermore, the IORW method assumes that the NDE, *β*_dir_, can be estimated from

(4) $$h^{-1}(E[Y\{a,M(a^{\prime })\}|C=c;\;\beta_{\text{dir}},\psi_{a^{\prime }}])={\tilde{\gamma }}_{\text{dir}}(c;\;\beta_{\text{dir}})a+{\tilde{\gamma }}^{a^{\prime }}(c;\psi_{a^{\prime }})$$

where ${\tilde{\gamma }}_{\text{dir}}(c;\beta_{\text{dir}})$ is a parametric model for *γ*_dir_(*c*) with unknown parameter *β*_dir_ and *β* = (*β*_dir_, *ψ*_*a′*_). The key result of Tchetgen Tchetgen (2013) is that, under consistency, conditional exchangeability and positivity, plus the assumption that model (4) is correctly specified, we can obtain an unbiased estimate of *β* by solving the population estimating equation

$$E\{U(\beta^{*})\}=0$$

where

$$U(\beta^{*})=\text{OR}(M,A|C{)}^{-1}\Delta_{\text{dir}}(A,C;\beta^{*})\{Y-b(A,C;\beta^{*})\}$$

where $b(a,c;\beta^{*})=h\{\tilde{\gamma }(c;\beta_{\text{dir}}^{*})a+{\tilde{\gamma }}^{a^{\prime }}(c;\psi_{a^{\prime }}^{*})\}$ for any β*, Δ_dir_ is defined similarly to Δ_tot_, and

(5) $$\text{OR}(M,A|C)=\frac{f_{M|A,C}(M|A,C)f_{M|A,C}(M=m_{0}|A=0,C)}{f_{M|A,C}(M=m_{0}|A,C)f_{M|A,C}(M|A=0,C)}\;\;=\frac{f_{A|M,C}(A|M,C)f_{A|M,C}(A=0|M=m_{0},C)}{f_{A|M,C}(A=0|M,C)f_{A|M,C}(A|M=m_{0},C)}$$

where *f*_*A∣M,C*_ is the conditional density of *A* given *M* and *C*, and *m*_0_ is a reference value for *M*. The equality in expression (5) comes from the fact that the odds ratio (OR) is symmetric and, since no distributional assumptions are placed on *f*_*A∣M,C*_, this OR parameterization is also valid for non-binary exposures. After estimating the total effect and NDE, the NIE (or joint NIE if there are multiple mediators) can be estimated by ${\hat{\gamma }}_{\text{tot}}-{\hat{\beta }}_{\text{dir}}$, and confidence intervals for all effects can be estimated by the non-parametric bootstrap or directly via variance–covariance matrix estimation. We refer readers who are interested in a more detailed description of the method to Tchetgen Tchetgen (2013).

Therefore, by using IORW, we can obtain an unbiased estimate of the NDE of an exposure on an outcome through an arbitrary number of mediators via weighted regression, with the inverse OR in equation (5) being used as the weights for those who are exposed (those who are unexposed receive a weight of 1). This is a very attractive feature of the IORW approach, since it means that it can easily handle multiple mediators through including additional terms in the OR model (one for each mediator), and continuous, binary and categorical mediators can all be included in the same model, if desired.

Applied to our research question and data, we first estimated stabilized IORW weights by fitting a logistic regression model for *diabetes mellitus* given brain hypoperfusion, white matter disease and covariates:

(6) $$\mathrm{logit}\{P(A=1|M_{1},M_{2},C)\}=\beta_{0}+\beta_{1}M_{1}+\beta_{2}M_{2}+\delta^{\prime }C.$$

Although we did not consider any interactive terms in our analysis because they were considered clinically unlikely or were found to be statistically non-significant, interactions could be considered in the specification of model (6) if they are believed to be present, since the statistical consistency of the final estimators for the NDE and NIE relies on a correctly specified OR model. Using model (6), we calculated a predicted OR for diabetes for each individual in our study by taking advantage of the invariance of the OR, which enabled us statistically to consider diabetes as the dependent variable and to treat brain hypoperfusion and white matter as independent variables even though our assumed causal relationship is that diabetes causes brain hypoperfusion and white matter disease. For those who were diabetic, we calculated an IORW by taking the inverse of their predicted OR and, for those who were not diabetic, we defined their IORW to be 1. As mentioned in Nguyen et al. (2015), more efficient estimates of the NDE and NIE can be obtained by using stabilized weights, which are obtained by multiplying each individual’s predicted OR by the odds of exposure when the mediators are set to their reference value, and then inverting the result. Equivalently, one could instead estimate the predicted odds of exposure by using model (6) instead of a predicted OR and then taking the inverse to obtain an inverse odds weight for each individual, and this is the stabilization method that we used.

After calculating inverse odds weights for each individual, we fitted a conditional logistic model for the total effect of diabetes on MCI or AD conditional on covariates:

(7) $$\mathrm{logit}\{P(Y=1|A,C)\}=\alpha_{0}+\alpha_{1}A+\gamma^{\prime }C.$$

The parameter *α*_1_ in model (7) is interpreted as our estimate of the total effect. We then fitted the same model, but used the inverse odds weights:

(8) $$\mathrm{logit}\{P(Y=1|A,C)\}={\tilde{\alpha }}_{0}+{\tilde{\alpha }}_{1}A+{\tilde{\gamma }}^{\prime }C.$$

Crucially, no mediator terms are included in this model. In model (8), the parameter ${\tilde{\alpha }}_{1}$ is interpreted as our estimate of the NDE. Finally, we obtained an estimate of the NIE by subtracting the NDE from the total effect:

(9) $$\text{NIE}=\alpha_{1}-{\tilde{\alpha }}_{1}.$$

Following other studies in the literature that used the IORW approach (see Nguyen et al. (2015)), we *a priori* used a cut-off of *p* = 0.10 and 90% confidence intervals (CIs) to determine statistical significance. Given that the IORW approach is known to produce larger standard errors (Nguyen et al., 2015), and because our research focus was not on the binary question of whether there is or is not a significant NIE (in favour of quantifying how large the NIE is), we felt that this decision was appropriate. To estimate 90% CIs around our estimates, we used 1000 non-parametric bootstrap replications of model (6)-(9). All analyses were implemented in Stata 15 (Stata Corp., 2017). Readers who are interested in pursuing a similar analysis to ours using the IORW approach are referred to Nguyen et al. (2015), where step-by-step details are given in the appendix.

## 4. Results

### 4.1. Primary and secondary outcomes

The demographic characteristics of the analytic sample are displayed in Table 1. Approximately 12.9% of our analytic sample was classified as having prevalent diabetes at baseline. Compared with non-diabetics, diabetics were significantly less likely to be white (*p* < 0.01), to have at least a high school education (*p* < 0.01) and to have at least one APOE-4 allele (*p* = 0.02). Diabetics also had significantly lower total cholesterol levels (*p* < 0.01), and they were more likely to be male compared with non-diabetics (*p* < 0.01). We observed no differences between diabetics and non-diabetics in terms of baseline age or smoking status. Among those who were eligible for inclusion in our study, the participants who were included in the analytic sample and those who were excluded because of missing data did not significantly differ on any of the covariates that were considered (*p* > 0.1).

Table 2 displays the results of our primary causal mediation analysis examining the relationship between diabetes and clinical cognitive impairment through brain hypoperfusion and white matter disease, as well as the two secondary analyses that examined MCI and dementia separately. When combining MCI and dementia as a single end point, we found that diabetes significantly increased the odds of MCI or dementia by 51% in total (OR = 1.51; 90% CI (1.22, 1.86); *p* < 0.01). This effect was 34% mediated by brain hypoperfusion (indirect effect *β* = 0.14 *versus* total effect *β* = 0.41). The direct effect of diabetes on cognitive impairment was significant at the *p* = 0.10 level (OR = 1.31; 90% CI (1.01, 1.72); *p* = 0.09), but the indirect effect was not (OR = 1.15; 90% CI (0.98, 1.35); *p* = 0.16).

Our analyses of the secondary outcomes of MCI only or dementia only followed the same patterns as the primary analysis. Specifically, we found that diabetes significantly increased the adjusted odds of incident MCI by 54% in total (OR = 1.54; 90% CI (1.21, 1.95); *p* < 0.01). This effect was 38% mediated by brain hypoperfusion and white matter disease (indirect effect *β* = 0.16 *versus* total effect *β* = 0.43). However, neither the direct nor the indirect effects were significant at the *p* = 0.10 level. Finally, our analysis with dementia alone as the end point suggested that diabetes significantly increased the odds of incident dementia by 56% in total (OR = 1.56; 90% CI (1.16, 2.11); *p* = 0.01), and this effect was 41% mediated by brain hypoperfusion and white matter disease (indirect effect *β* = 0.18 *versus* total effect *β* = 0.45). Neither the direct nor the indirect effects were significant on their own.

### 4.2. Sensitivity analyses

To assess our causal assumptions, we conducted several sensitivity analyses (Table 3). For convenience, all sensitivity analyses focused only on the primary outcome and CIs of estimates. First, we assessed how changing our definition of white matter disease would affect our results by changing the cut-off for disease by one point in either direction, and we found, not surprisingly, that raising the threshold weakened the indirect effect. Next, we changed our model specifications from logistic regression to Poisson regression while keeping the covariates in the model the same. As expected, we found that the risk ratios that were estimated by Poisson regression were closer to the null than the ORs that were estimated by logistic regression; however, the NDE and NIE estimates were qualitatively similar to each other. We also ran several additional sensitivity analyses in which we changed the definition of level of education, changed the model specification to include interaction terms between diabetes status and age, sex and race, or changed the model specification to include non-linear terms for cholesterol level, age and body mass index. In all these sensitivity analyses, our NDE and NIE estimates were fairly consistent (the results are not shown). Finally, we assessed how our NDE and NIE estimates would change if we used another mediation approach that also handles multiple mediators and estimates joint mediated effects—namely using weighted parametric regression models (VanderWeele and Vansteelandt, 2014) and natural effects models (Steen et al., 2017b) (Table 4). The method of VanderWeele and Vansteelandt (2014) produced similar results to the IORW approach; however, the natural effects modelling approach yielded a noticeably higher NDE estimate, and therefore noticeably lower NIE estimate, compared with the IORW approach.

We also ran several sensitivity analyses that were related to the key set of assumptions that we made regarding no unmeasured confounding. To assess the possibility of unmeasured confounding by omitted observed variables, we included five additional covariates in our model as potential confounders of the relationships between diabetes, brain hypoperfusion, white matter disease and/or cognitive impairment:

- a baseline log_10_(interleukin-6 level),
- baseline log_10_(C-reactive protein level),
- depressive status (measured via the Center for Epidemiological Studies depression scale CES-D),
- income level and
- physical activity (measured via the self-reported number of blocks walked in the month before baseline).

The inclusion of these additional covariates made the estimated NIE slightly larger, while also making both the NDE and the NIE non-significant at the *p* = 0.10 level (Table 3). We also ran a sensitivity analysis for unmeasured confounding by applying methods that were recommended by Tchetgen Tchetgen and Shpitser (2012). This method involves specifying a bias function for direct and indirect effects induced by unobserved confounding and iterating over a range of plausible values to see how the study results may change in the presence of various levels of unobserved confounding bias. For convenience, we ran the sensitivity analysis on participant 3MS scores rather than their adjudicated dementia status. We aimed to capture the possible bias that would result if those with diabetes who experienced brain hypoperfusion or white matter disease had, on average a potential 3MS score that was between 4 points lower and 4 points higher compared with those who had diabetes but did not experience brain hypoperfusion or white matter disease. These end points were chosen to capture meaningful and plausible values for differences in CHS participants’ cognitive performance. We observed that the direct effect and indirect effects were fairly robust to our specified levels of unmeasured confounding.

## 5. Discussion

### 5.1. Biological and clinical mechanisms

With respect to the primary goal of our analysis, we found that *diabetes mellitus* significantly increased the odds of clinical cognitive impairment by using both MCI and dementia outcomes, and that brain hypoperfusion and white matter disease mediated over a third of the total effect of diabetes on MCI and dementia. When MCI and dementia were considered separately, we found similar patterns to those with the joint outcome, with the indirect effect estimate being slightly stronger than the direct effect estimates in both cases. In general, our results fit within the broader literature showing that diabetics have a significantly greater risk of accelerated cognitive decline and cognitive impairment compared with non-diabetics (Cheng et al., 2012; Cukierman et al., 2005; Mayeda et al., 2015; Palta et al., 2017). Other studies have demonstrated that diabetics have higher cardiovascular burden, including brain lesions, that can possibly co-occur with cognitive impairment (Falvey et al., 2013; Kooistra et al., 2013; Luchsinger et al., 2007; Manschot et al., 2006; Singh et al., 2013). The study that comes closest to our own in terms of goals and result is Wang et al. (2017), which reported a significant mediating effect of brain pathology on the relationship between cardiovascular burden and cognitive decline; however, few details are available about how exactly the mediation analysis was conducted, and it is likely that they assumed strictly linear relationships between variables. Our results expand on these studies by directly evaluating this pathway by imposing a strict temporal ordering on events, and formally decomposing the effect of diabetes into both direct and indirect pathways through brain hypoperfusion and white matter disease

Our study advances epidemiologic approaches to the study of cognitive impairment in several ways. First, we used methods that enabled us to estimate more closely how a mechanistically established pathway in animal models may also be present in humans. Our study is also one of the first, if not the first, study to examine the full hypothesized pathway in humans between diabetes, adjudicated cerebrovascular burden and adjudicated cognitive impairment in a national cohort of older adults followed for over a decade. By capitalizing on the advantages of causal mediation analysis and the CHS, we could study the pathway between diabetes and cognitive impairment through brain hypoperfusion and white matter disease in a precise temporal order. Finally, to our knowledge, this is the first study to use the IORW approach to causal mediation analysis to study a research question in clinical cognitive impairment.

At the same time, whereas we did see a statistically significant ACE and fairly sizable NDE and NIE estimates, we failed to see a significant NIE of diabetes on cognitive impairment through brain hypoperfusion and white matter disease at our *a priori* level of *α* = 0.10 and, if we had used the conventional *α* = 0.05 threshold, we would have failed to see a significant NDE as well (Table 2). When we repeated our analysis by using the alternative methods of VanderWeele and Vansteelandt (2014) and Steen et al. (2017b), we found similar results; however, the estimated NIE was smaller than originally estimated with IORW. Taken together, it is reasonable to be cautious when interpreting our findings. For example, although our proportion mediated was approximately a third in the main analysis, the bootstrap 90% confidence interval for this proportion is (−0.32, 0.98). Because this covers essentially every possible proportion, it is less clear what the true proportion mediated is. Another related limitation is that the proper application of mediation analysis requires that the exposure precede the mediator, which in turn precedes the outcome of interest. In our study, this important requirement meant that 10% of the total cognition study cohort was ineligible for inclusion because of our analytic exclusion criteria. It also meant that we could not examine the mediating role of other common cardiovascular factors, like hypertension, because doing so would result in losing 40–50% of the otherwise eligible sample who had a history of hypertension at baseline and would threaten our ability to detect significant effects even when they are truly present. Finally, prior studies suggest that midlife diabetes and cardiovascular risk factors are just as important, if not more important, than late life diabetes and cardiovascular risk factors (Debette et al., 2011; Gottesman et al., 2014; Rawlings et al., 2014; Tuligenga et al., 2014; Whitmer et al., 2007). In the CHS, participants were past midlife, and almost all information about cardiovascular risk factors was measured in late life. Although this does not invalidate any of the results of our study, caution should be exercised when interpreting our results in the context of the entire life course, as there are likely to be many complicated mechanisms at play and our study focused on a small subset of these.

### 5.2. Choice of the inverse odds ratio weighting method

When the goal of a study is to conduct a mediation analysis with multiple mediators, several options are available. However, the IORW approach has several key advantages over other comparable methods. For example, other multiple-mediator methods require that a joint density for the mediators be specified (Hong and Nomi, 2012; Lange et al., 2014) or impose restrictions, like sequential causal ordering (Daniel et al., 2015). The IORW approach avoids these potential complexities through the use of OR weights. Because the IORWs are calculated from a single model of the exposure given the mediators and covariates, it can handle multiple mediators in an easy straightforward way by simply including a term for each desired mediator. Another advantage of the IORW approach is that it does not require a model for the outcome given the exposure, mediator and covariates. Consequently, it is equally valid in the presence or absence of exposure–mediator interactions, unlike comparable weighting methods (e.g. the approaches of VanderWeele and Vansteelandt (2014) and Steen et al. (2017b) that were used in our sensitivity analyses) that require that exposure–mediator interactions be correctly specified whenever present.

These advantages do come at a cost. Whereas other methods can produce path-specific effects in certain situations (Daniel et al., 2015; Lange et al., 2014; Steen et al., 2017a), the IORW approach cannot. Another disadvantage of the IORW approach is that it can produce larger variance estimates compared with other methods, which can make detecting smaller indirect effects more difficult in smaller samples (see Nguyen et al. (2015)). Finally, although the IORW approach requires fewer models than other mediation approaches, it still requires the correct specification of both the total effect and the OR weight model for the mediation effects to be unbiased (i.e. care needs to be taken to determine whether model (6) is correct, possibly through positing alternative models and checking how they compare and how robust the results are to model changes).

Given these advantages and disadvantages, we selected the IORW approach over other methods, primarily because of its flexibility and straightforwardness. Although it does require correct model specifications, this is a standard requirement for all methods. Since the IORW approach requires fewer models and the models that we posit are simpler objects than conditional densities, the chances of model misspecification are relatively lower compared with other methods. It would have been possibly useful to have the ability to estimate path-specific effects; however, we believe that this downside did not outweigh the other advantages that the IORW approach had for our research question.

### 5.3. Causal assumptions: feasibility and sensitivity analysis

To interpret our results as causal effects, it is important to assess how reasonable the assumptions that were described earlier are for our study. The more plausible the assumptions are to hold, the more confident we can be that the effects that are estimated are causal. With respect to the positivity assumption, we did find that there was a minor amount of non-overlap in some individual covariate distributions (e.g. age) between those with diabetes *versus* not, and it is possible that there were certain covariate combinations that also were unique to either the diabetes group or the control group. However, we do not feel that these practical positivity violations considerably altered our results.

Our consistency assumption is somewhat more controversial. A common consistency violation occurs when there are multiple versions of the treatment, which makes counterfactuals ill defined. When the treatment is a health condition, like diabetes, one could imagine that there are multiple versions of the health condition (e.g. diabetes due to not exercising could be one version, and diabetes due to eating a poor diet could be another version), which would mean that consistency is questionable. However, there have been some references on this assumption that argue that what matters is whether or not the possibly multiple versions of treatment result in the same outcome (Cole and Frangakis, 2009; Pearl, 2010; VanderWeele, 2009), and we believe that it is reasonable to assume that it is the diabetes itself that impacts one’s future risk for brain hypoperfusion, white matter disease and MCI or dementia and that how one developed diabetes is not relevant (i.e. we assume treatment variance irrelevance, which is then used as part of the consistency assumption).

Regarding the no-interference assumption, we believe that this is reasonable. Violations of this assumption are more common in studies where the treatment is a vaccine or educational programme and/or where there is social interaction between participants because they are room mates, schoolmates, friends, neighbours, family, etc. (Ogburn and VanderWeele, 2014; VanderWeele et al., 2014). Although some CHS participants did come from the same household, which would possibly violate this assumption, we believe that any possible interference effects would not have invalidated our results.

The last two general (i.e. not specific to mediation) causal assumptions—no measurement error and correct model specification—are somewhat less likely to hold. In any study involving socio-economic or clinical measurements, it is almost impossible to measure covariates with 100% accuracy, which means that the no measured confounding assumption is very likely to be violated. However, the CHS devoted many resources towards collecting accurate data. For example, the CHS diagnosed individuals with diabetes on the basis of objective blood biomarkers and pharmacy records instead of self-report. However, with respect to our exposure, the CHS definition of diabetes was established in a younger population than the CHS recruited; therefore, it may be that our definition of diabetes, which is valid in young or middle-aged adults, is somewhat mismeasured in older adults. With respect to our mediators and outcome, cognitive impairment status and all cardiovascular-related events were adjudicated by a panel of experts. Although these do not completely rule out measurement error, it is less likely than if the CHS had relied more heavily on self-reported health variables (Butler et al., 1987). As described in Section 4, we also ran several sensitivity analyses related to how variables were defined and how our models were specified, and we failed to see any notable changes from changing how variables were defined or changing the link function of our models.

With respect to our unmeasured confounding assumptions that are specific to mediation analysis, we believe that they are also plausible; however, it is impossible for us to know truly. Since we are considering a joint mediator, we are assuming that there is a set of covariates *C* such that assumptions 1–4 are satisfied for brain hypoperfusion and white matter disease jointly. To assess assumptions 1–3, we performed a sensitivity analysis in which we added five extra covariates to the model. We found that our estimated effects did change slightly, and our direct effect estimates became insignificant at the *α* = 0.10 level. In our opinion, this means that these covariates may have been confounders that should have been included in our main model; however, it may also be that these variables are not confounders and adjusting for them either introduced bias (by opening up new collider paths or closing relevant causal paths) or unnecessarily increased our variances, thereby making the estimates non-significant. It is also plausible that there are truly unmeasured confounders of the exposure–mediator, mediator–outcome or exposure–outcome relationship(s) that we failed to include in our models, simply because the CHS did not collect them. In our opinion, however, we did manage to adjust for most, if not all, of the relevant confounders.

Finally, the cross-world assumption 4 is impossible to test empirically because it would involve setting *A* to two different values simultaneously. However, we cautiously believe that this assumption is plausible in our setting. The cross-world assumption will be violated if either of two scenarios happen:

- there is at least one variable *L* that is a mediator–outcome confounder that lies on the causal pathway between *A* and *M* (VanderWeele, 2011) or
- there is an unmeasured variable *U* that only exists as a mediator–outcome confounder under cross-world treatment assignments (Robins and Richardson, 2010).

With regard to the latter scenario, it is difficult to imagine such a confounder existing for cognitive impairment and brain hypoperfusion and white matter disease, so we believe that it does not apply. With regard to the possibility that there is at least one mediator–outcome confounder affected by exposure, we cannot be certain that this does not apply. For example, it could be that diet is such a confounder, since diabetics could change their diet after their diagnosis, and diet has been shown to be associated with both cerebrovascular pathology and cognitive impairment (Esposito et al., 2010; Psaltopoulou et al., 2013). To assess how a violation of the cross-world assumption would affect our results, we used the sensitivity analysis technique of Tchetgen and Shpitser (2012) which involves specifying various levels of unmeasured confounding bias (from, for example, omitting an unmeasured mediator–outcome confounder) and iterating over them to see how the estimates of the NDE and NIE would change as a function of this bias (see the appendix of Nguyen et al. (2015) for an accessible step-by-step description of this sensitivity analysis). We found that under realistic amounts of bias our results did not change. This supports the belief that our results are robust against a possible cross-world assumption, even though we cannot fully rule it out.

### 5.4. Future directions

Since accelerated cognitive aging and incident cognitive impairment are believed to be a complex phenomenon with possibly many mediating pathways between exposures and cognitive outcomes, this paper has shown that methods such as IORW can be useful in determining the combined relative contribution of certain hypothesized mediators. Several follow-up studies could be done motivated by our results. First, these findings merit replication in other longitudinal studies that began in midlife with similar measures and are on going, like the Baltimore Longitudinal Study of Aging (Ferrucci, 2008) and the Atherosclerosis Risk in Communities Study (ARIC Investigators, 1989). These additional data could address possible pathways between midlife risk factors and late life cardiovascular and neurocognitive health, which is not possible in the CHS. Second, it will be important to examine path-specific effects of diabetes on cognitive impairment. In our study, we estimated the mediated effect of both the brain hypoperfusion and white matter disease pathways together. However, there are methods that compute path-specific effects (Avin et al., 2005; Miles et al., 2017; Steen and Vansteelandt, 2019) that could reveal which pathway may be stronger (or not) when it comes to diabetes and cognitive impairment. Specifying the contributions of the vascular and insulin pathways to cognitive impairment will be increasingly valuable as the field strives to identify the combination of modifiable factors to prevent or delay cognitive impairment best and to improve quality of life as the population shifts towards being older.

## Figures and Tables

**Table 1.** Demographic characteristics of the CHS Cognition Study eligible sample (*N* = 3225)

Characteristic	Results for non-diabetics, N = 2810	Results for diabetics, N = 415	p-value
Baseline age†, mean (standard deviation)	71.8 (4.8)	72.0 (4.7)	0.61
White, *N* (%)	2463 (87.7)	314 (75.7)	< 0.01
Male sex, *N* (%)	1099 (39.1)	203 (48.9)	< 0.01
High school educated‡, *N* (%)	2179 (77.5)	289 (69.6)	< 0.01
Total cholesterol§, mean (standard deviation)	212.6 (37.4)	203.1 (41.8)	< 0.01
> 1 APOE-4 allele, *N* (%)	635 (24.6)	71 (18.9)	0.02
Current smoker, *N* (%)	310 (11.0)	40 (9.6)	0.39
Former smoker, *N* (%)	1171 (41.7)	168 (40.5)	0.71
CES-D-score§§	4.2 (4.1)	4.9 (4.8)	< 0.01

† Age was measured in years.

‡ High school educated was defined as completing 12 years of education *versus* not.

§ Total cholesterol was measured in units of milligrams per decilitre.

§§ CES-D ranged from 0 to 29. A score of 8 or more indicates the presence of depressive symptoms.

**Table 2.** Results of causal mediation analysis

	*Mediators: brain hypoperfusion and white* *matter disease*		
	*OR*	*90% CI*	*p-value*
MCI or dementia†			
Total effect	1.51	(1.22, 1.86)	< 0.01
Direct effect	1.31	(1.01, 1.72)	0.09
Indirect effect	1.15	(0.98, 1.35)	0.16
MCI‡			
Total effect	1.54	(1.21, 1.95)	< 0.01
Direct effect	1.31	(0.98, 1.75)	0.13
Indirect effect	1.18	(0.99, 1.40)	0.12
Dementia§			
Total effect	1.56	(1.16, 2.11)	0.01
Direct effect	1.30	(0.87, 1.94)	0.28
Indirect effect	1.20	(0.91, 1.58)	0.28

† A total of *N* = 2872 CHS participants were included in this analysis, of which 842 (29.3%) were classified as having MCI or dementia.

‡ A total of *N* = 2511 CHS participants were included in this analysis, of which 481 (19.2%) were classified as having MCI.

§ A total of *N* = 2391 CHS participants were included in this analysis, of which 361 (15.1%) were classified as having dementia.

**Table 3.** Model-based sensitivity analyses

	*Mediators: brain hypoperfusion and white matter* *disease*			
	*OR*	*90% CI*	*Risk ratio*	*90% CI*
Model 1†				
Total effect	1.51	(1.22, 1.86)	1.27	(1.13, 1.42)
Direct effect	1.31	(1.01, 1.72)	1.20	(1.03, 1.39)
Indirect effect	1.15	(0.98, 1.35)	1.06	(0.97, 1.16)
Model 2‡				
Total effect	1.51	(1.22, 1.86)	1.27	(1.13, 1.42)
Direct effect	1.34	(1.02, 1.75)	1.20	(1.04, 1.40)
Indirect effect	1.13	(0.96, 1.32)	1.05	(0.96, 1.15)
Model 3§				
Total effect	1.51	(1.22, 1.86)	1.27	(1.13, 1.42)
Direct effect	1.38	(1.05, 1.81)	1.22	(1.05, 1.42)
Indirect effect	1.09	(0.93, 1.29)	1.04	(0.95, 1.14)
Model 4§§				
Total effect	1.54	(1.22, 1.95)	1.27	(1.12, 1.45)
Direct effect	1.30	(0.97, 1.75)	1.18	(1.00, 1.39)
Indirect effect	1.18	(0.99, 1.42)	1.08	(0.98, 1.20)

† Model inputs were kept the same as in the original analysis.

‡ Model inputs were kept the same as in the original analysis, except that the white matter disease cut-off changed to greater than 1 out of 9 points.

§ Model inputs were kept the same as in the original analysis, except that the white matter disease cut-off changed to greater than 3 out of 9 points.

§§ Model inputs were kept the same as in the original analysis, plus additional terms for baseline log_10_(interleukin-6 level), baseline log_10_(C-reactive protein level) depressive symptoms, income level and physical activity.

**Table 4.** Mediation results estimated with alternative causal mediation methods

	*Mediators: brain hypoperfusion and white matter disease*					
	*IORW*		*Parametric* *regression*†		*Natural effects* *models*‡	
			
	*OR*	*90% CI*	*OR*	*90% CI*	*OR*	*90% CI*
MCI or dementia						
Direct effect	1.31	(1.01, 1.72)	1.40	(1.13, 1.66)	1.46	(1.18, 1.80)
Indirect effect	1.15	(0.98, 1.35)	1.11	(1.0, 1.23)	1.04	(1.00, 1.07)

† Estimates were calculated according to the method described in VanderWeele and Vansteelandt (2014).

‡ Estimates were calculated according to the method described in Steen and Vansteelandt (2019).
